# Supplementary material for: Environmentally adaptive MOF-based device enables continuous self-optimizing atmospheric water harvesting
Source: Nat Commun. 2022 Aug 19;13:4873. doi: 10.1038/s41467-022-32642-0 (PMC9391386; doi:10.1038/s41467-022-32642-0)
Supplement: Supplementary file 1 — Supplementary Information [file 41467_2022_32642_MOESM1_ESM.pdf]

Supplementary Information

Environmentally adaptive MOF-based device enables continuous self-optimizing atmospheric water harvesting

Husam A. Almassad,[1] Rada I. Abaza,[1] Lama Siwwan,[1] Bassem Al-Maythalony,[1] and Kyle E. Cordova[1,*]

[1]Materials Discovery Research Unit, Advanced Research Centre, Royal Scientific Society, Amman 11941, Jordan

*Corresponding author: kyle.cordova@rss.jo

This file includes:

Supplementary Figures 1 to 33

Supplementary Tables 1 to 15

Supplementary Notes 1 to 8

Supplementary References 1 to 7

Table of Contents

1. Supplementary Figures……………………………..………………………………….. 2

2. Supplementary Tables…...………………………………………………..………………… 25

3. Supplementary Notes..……………………………………………………………………. 35

4. Supplementary References………………...…………………………...……………… 44

1. Supplementary Figures

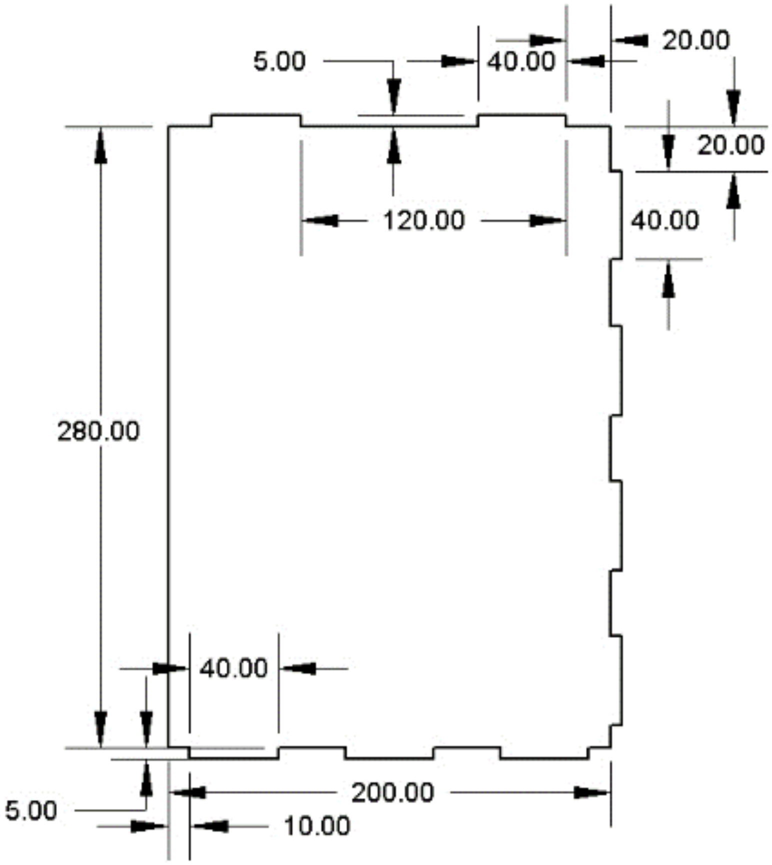

Supplementary Figure 1. Air intake compartment side wall.

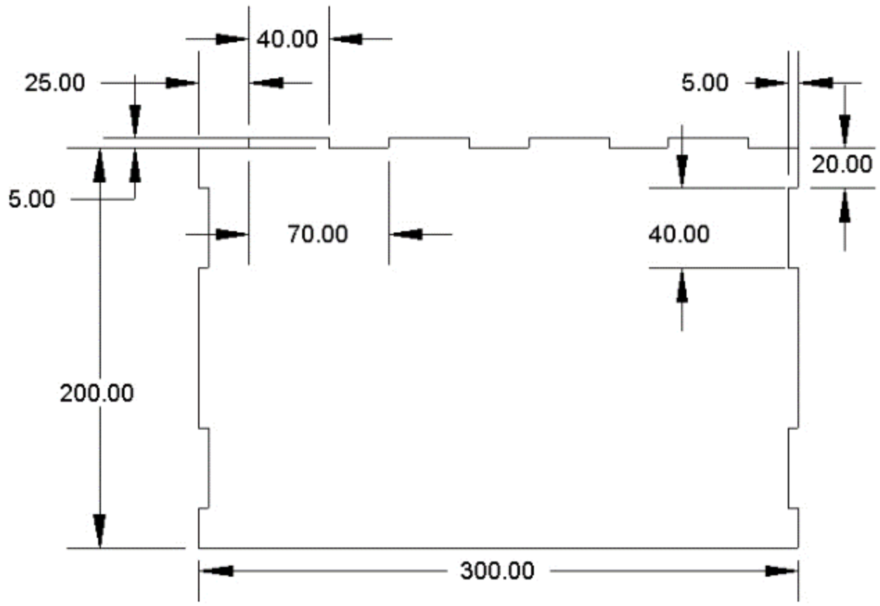

Supplementary Figure 2. Air intake compartment top wall.

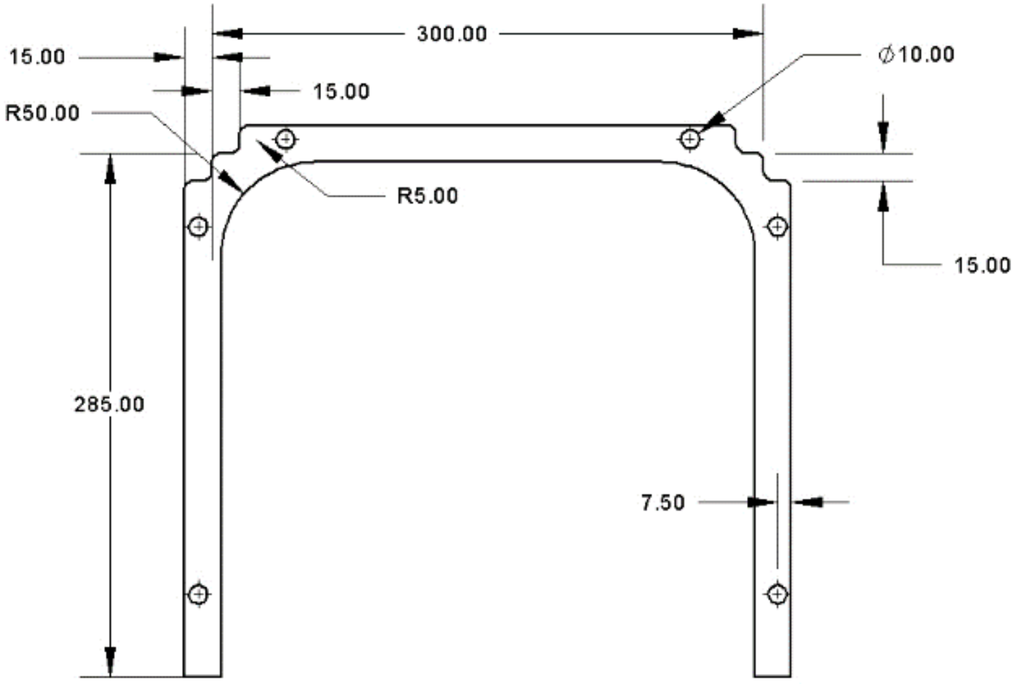

Supplementary Figure 3. Air intake compartment door frame.

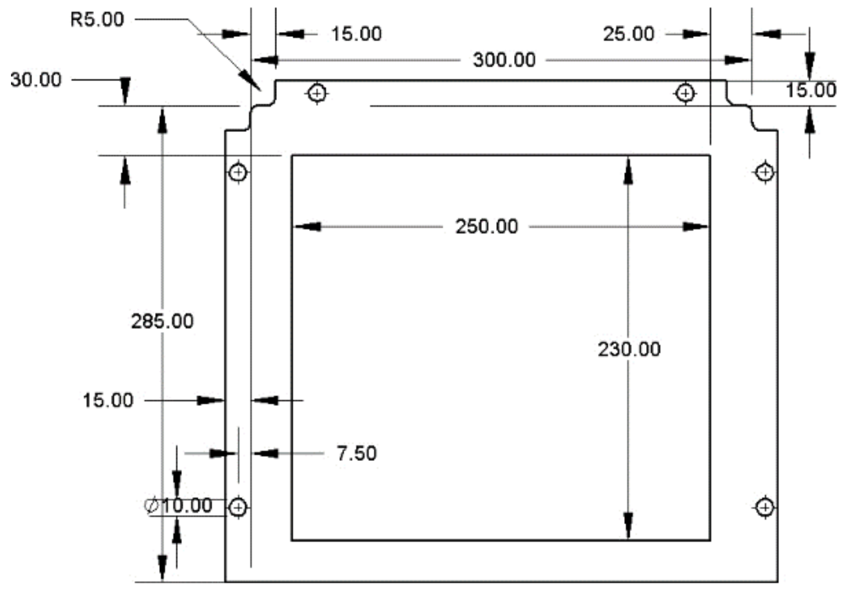

Supplementary Figure 4. Air intake compartment door with filter window.

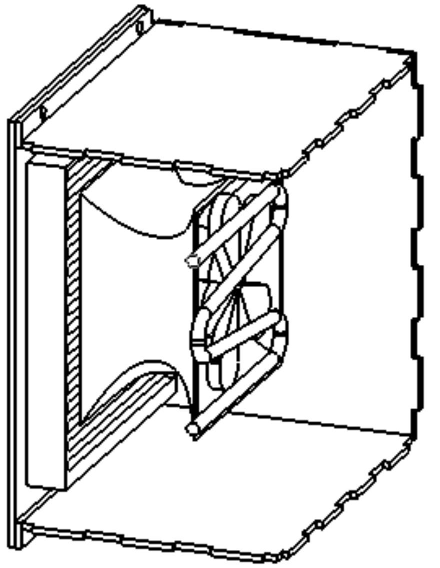

Supplementary Figure 5. Air intake compartment assembly.

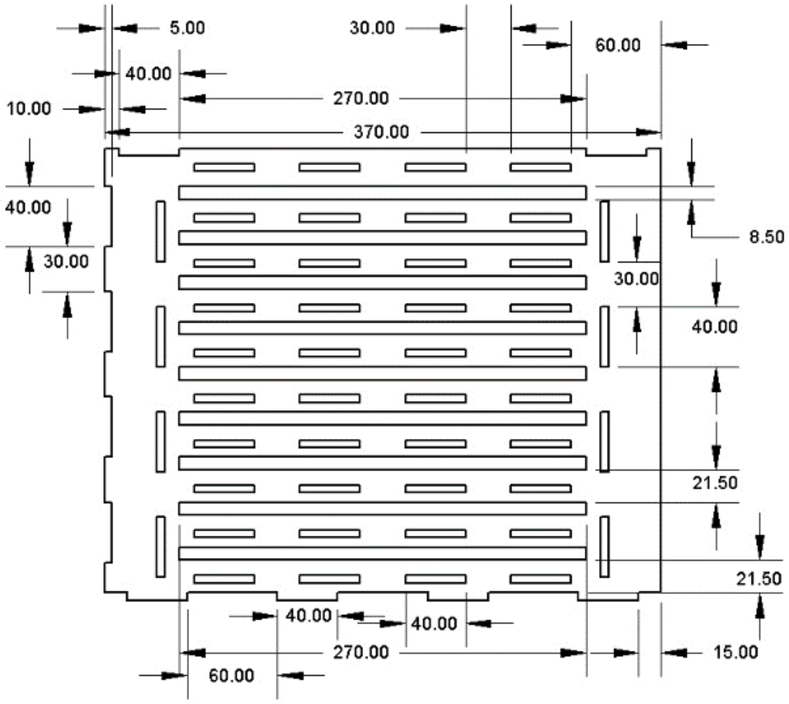

Supplementary Figure 6. Sorption compartment side wall.

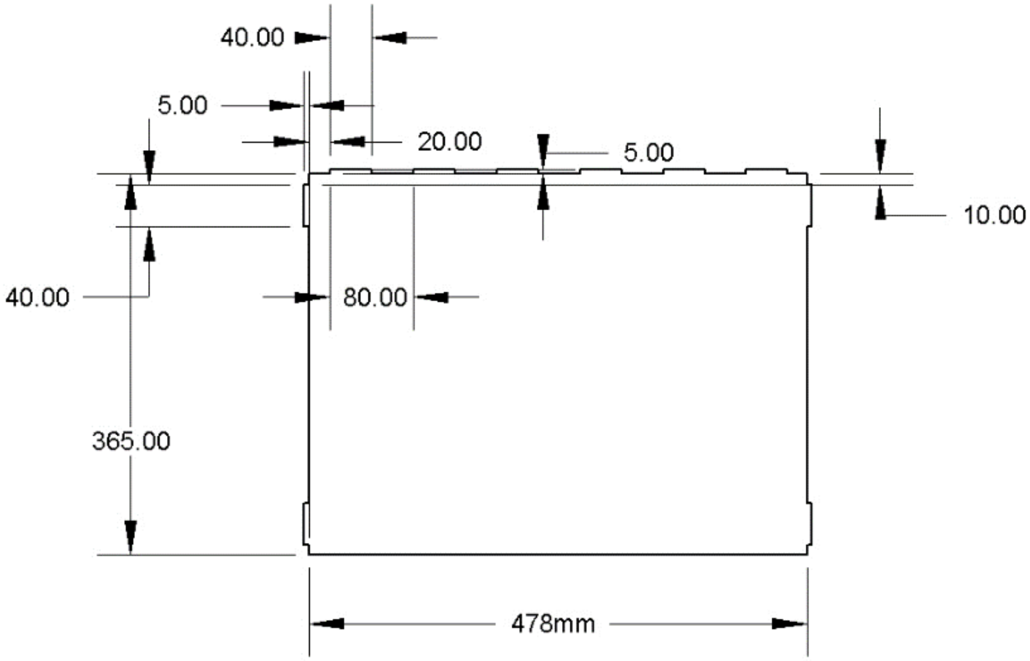

Supplementary Figure 7. Sorption compartment top wall.

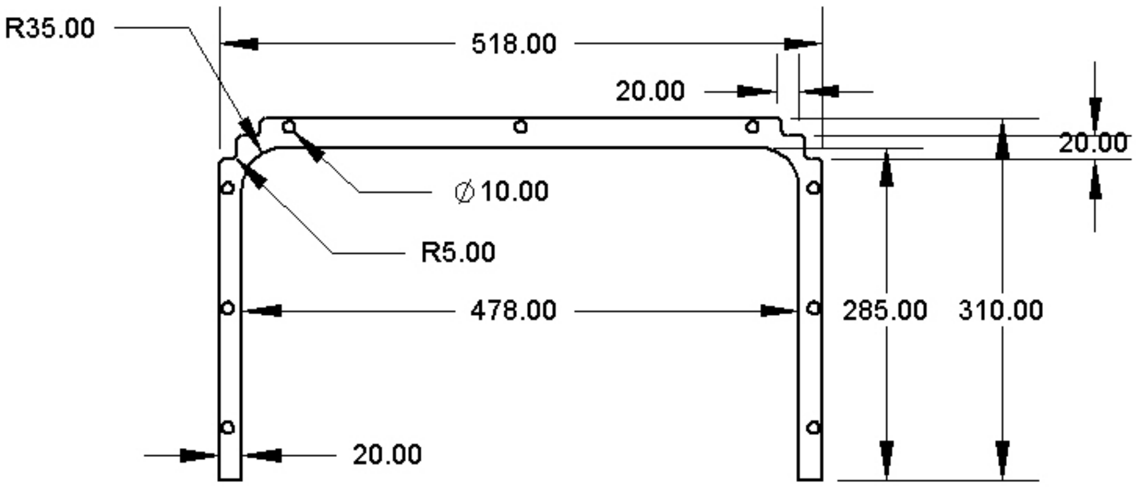

Supplementary Figure 8. Sorption compartment door frame.

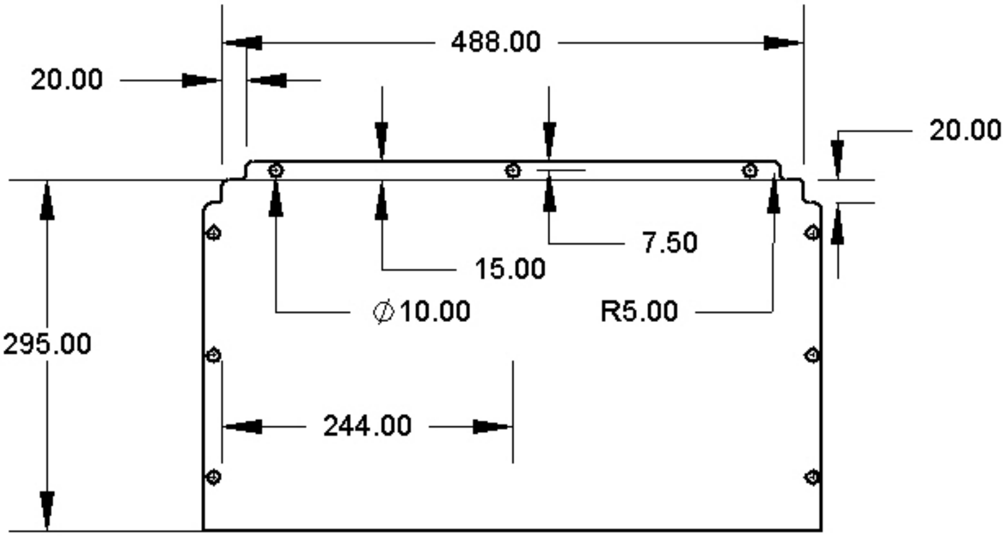

Supplementary Figure 9. Sorption compartment door.

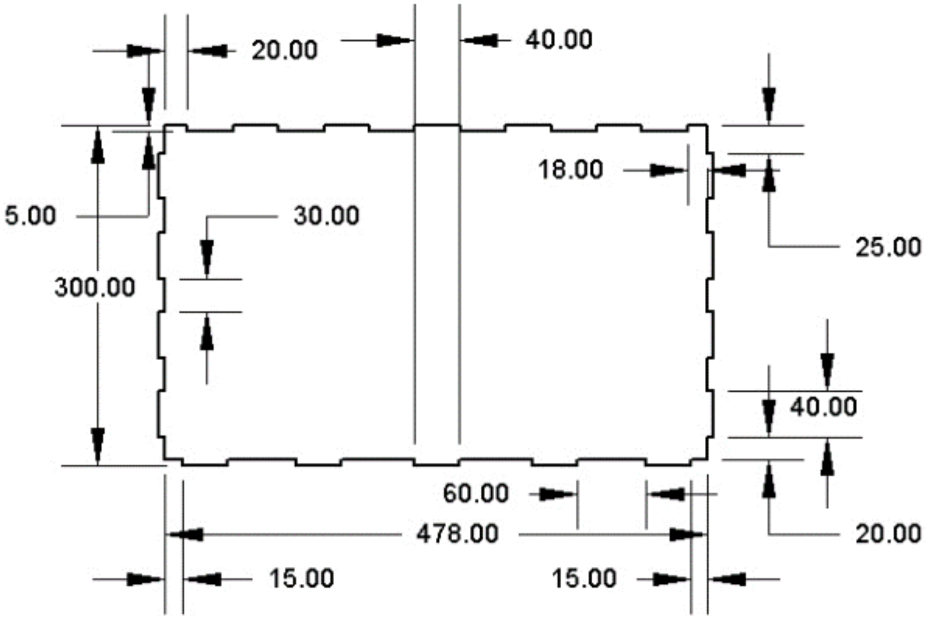

Supplementary Figure 10. Sorption compartment back side.

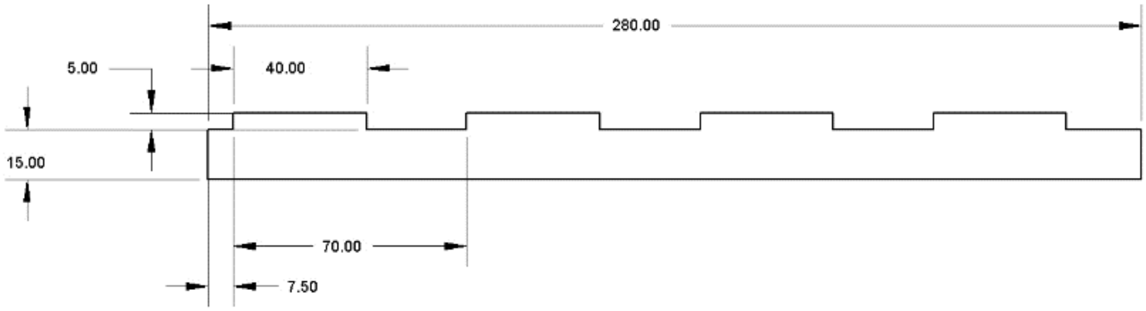

Supplementary Figure 11. Sorption compartment layer tray.

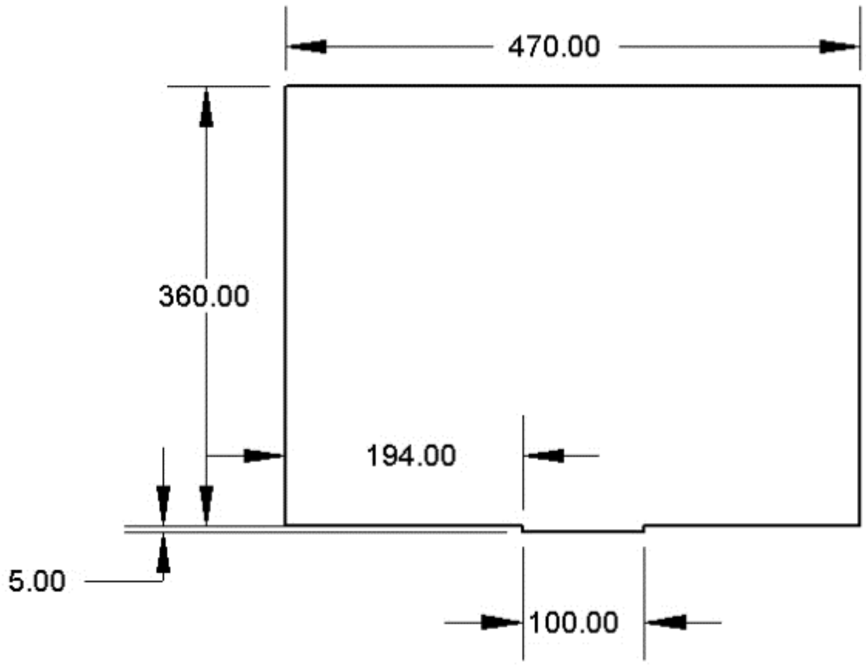

Supplementary Figure 12. Sorption compartment layer.

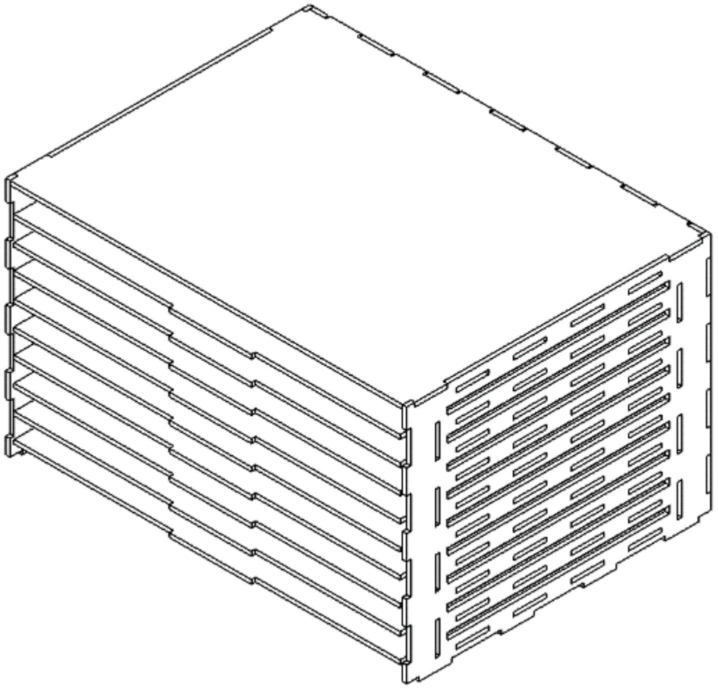

Supplementary Figure 13. Sorption compartment assembly.

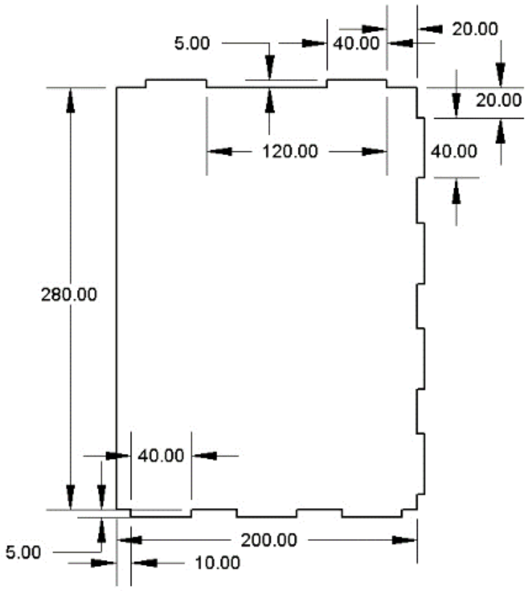

Supplementary Figure 14. Condensation compartment side wall.

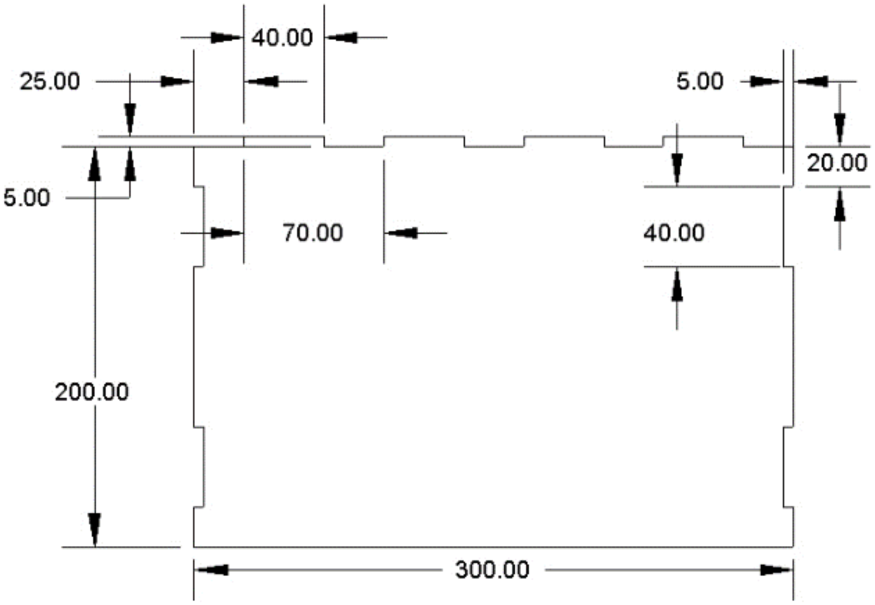

Supplementary Figure 15. Condensation compartment top wall.

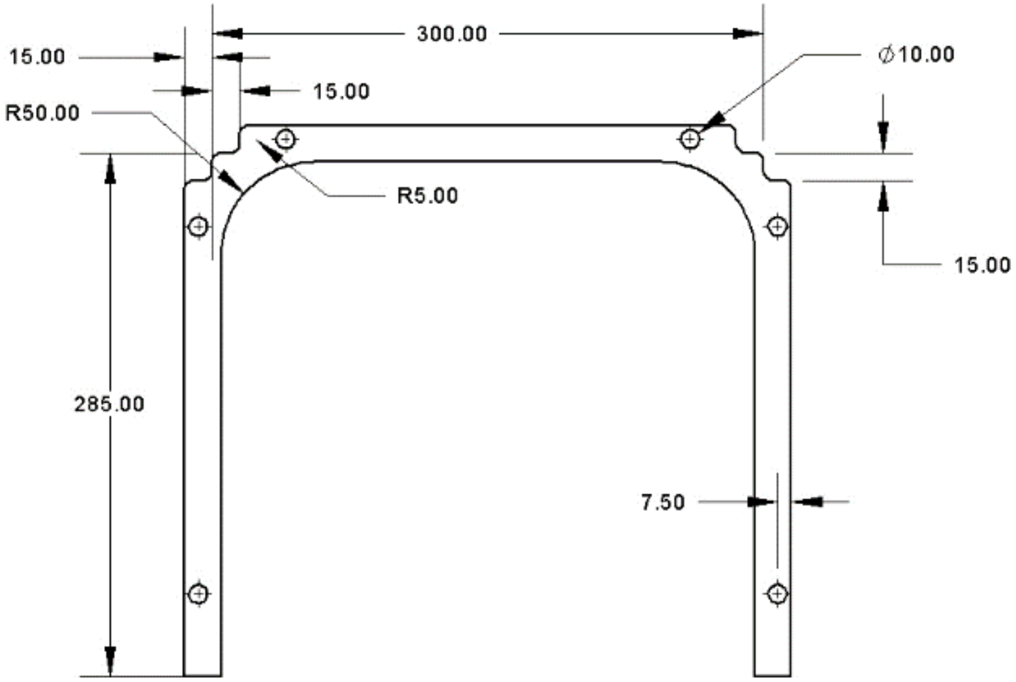

Supplementary Figure 16. Condensation compartment door frame.

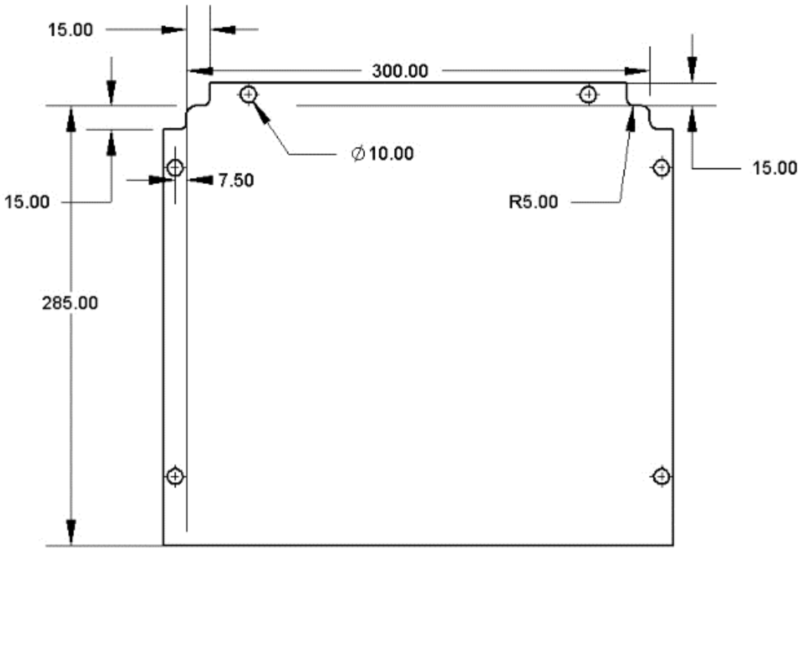

Supplementary Figure 17. Condensation compartment door.

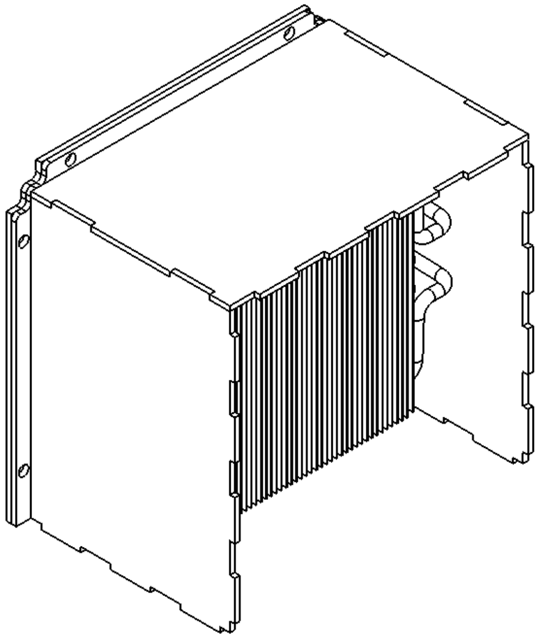

Supplementary Figure 18. Condensation compartment assembly.

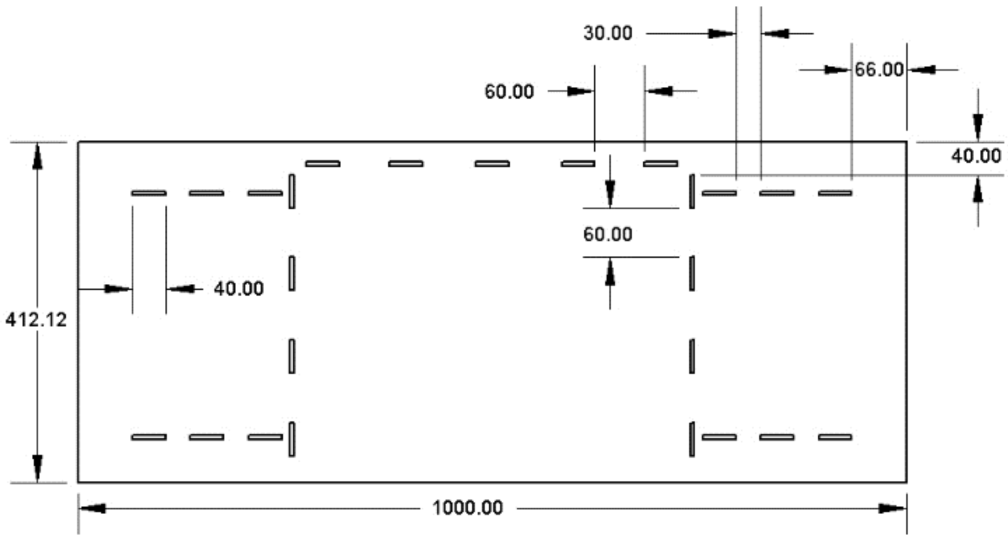

Supplementary Figure 19. Water harvester device base.

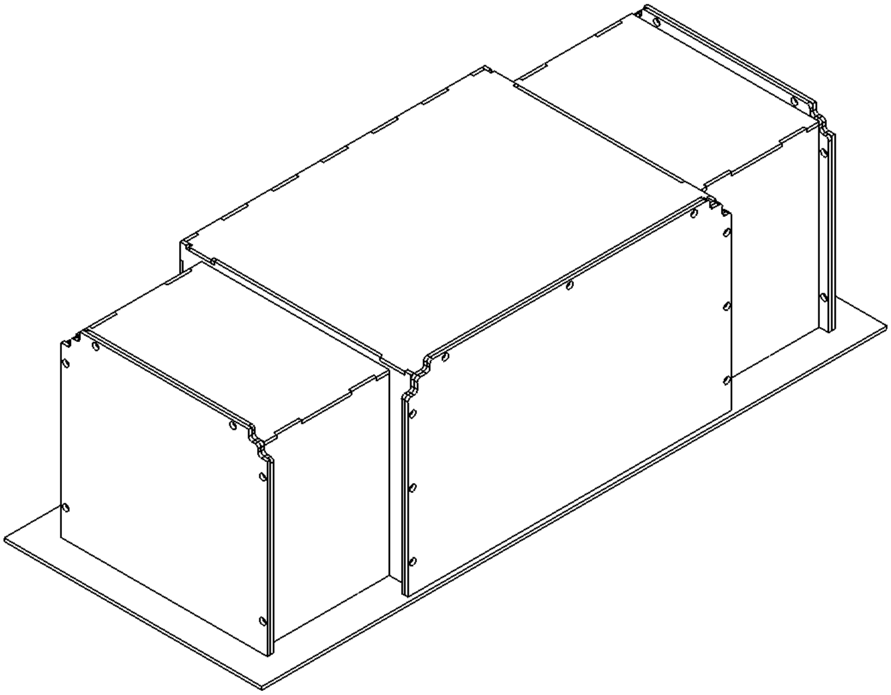

Supplementary Figure 20. Water harvester device full assembly.

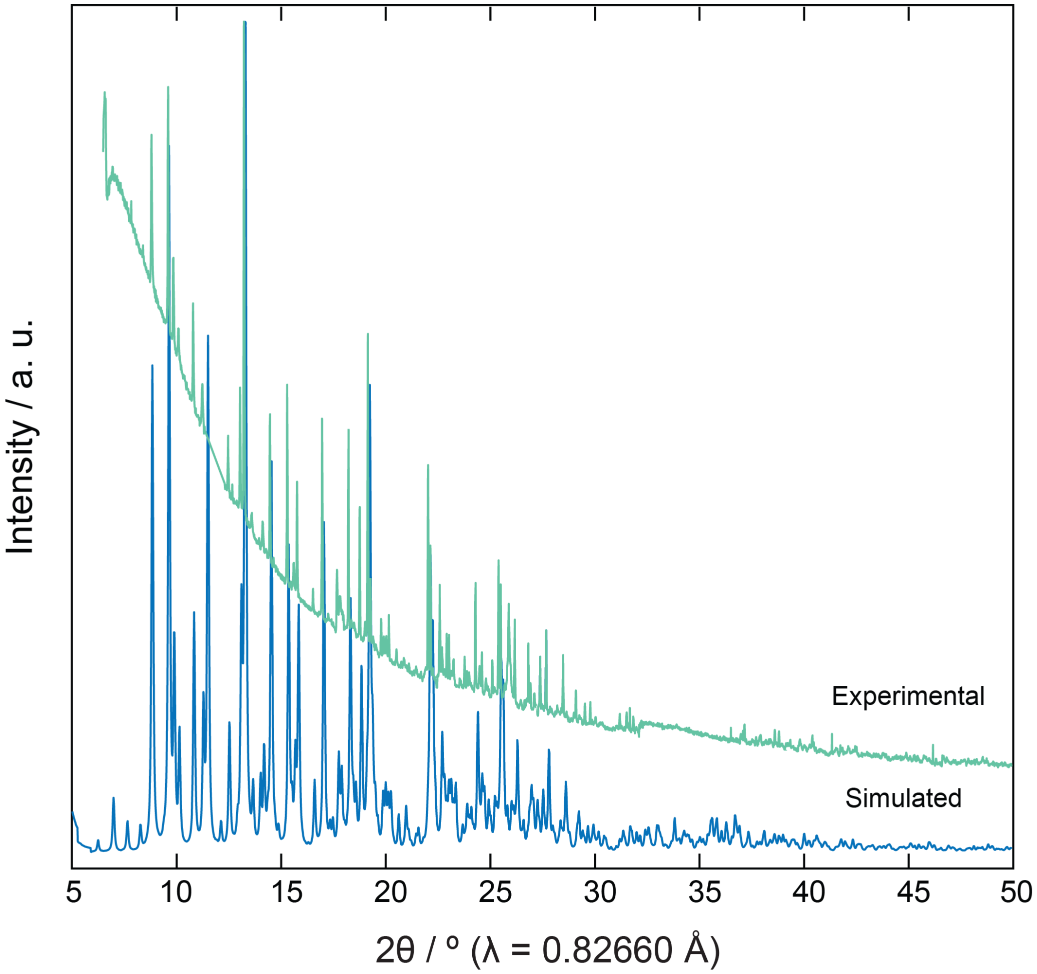

Supplementary Figure 21. PXRD of MOF-801 using synchrotron radiation.

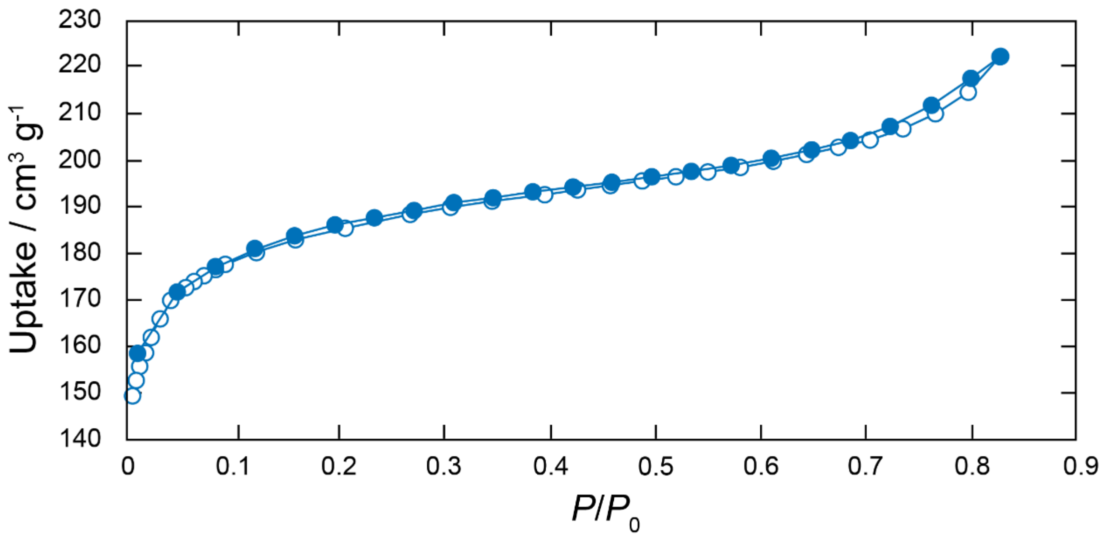

Supplementary Figure 22. N_2 adsorption isotherm at 77 K.

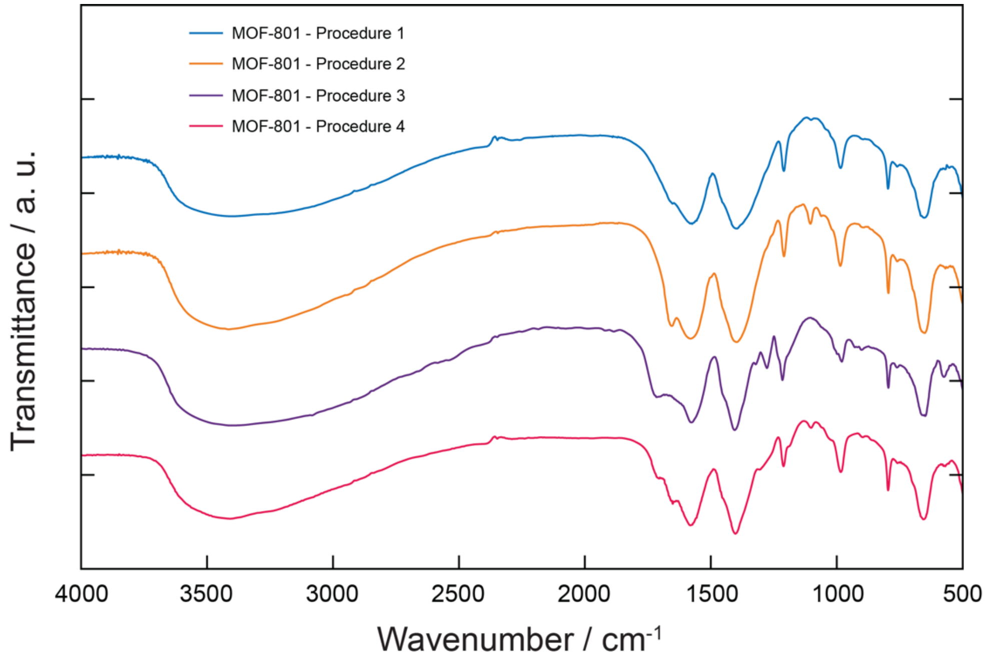

Supplementary Figure 23. FT-IR spectra for the MOF-801 synthesized from the four different procedures.

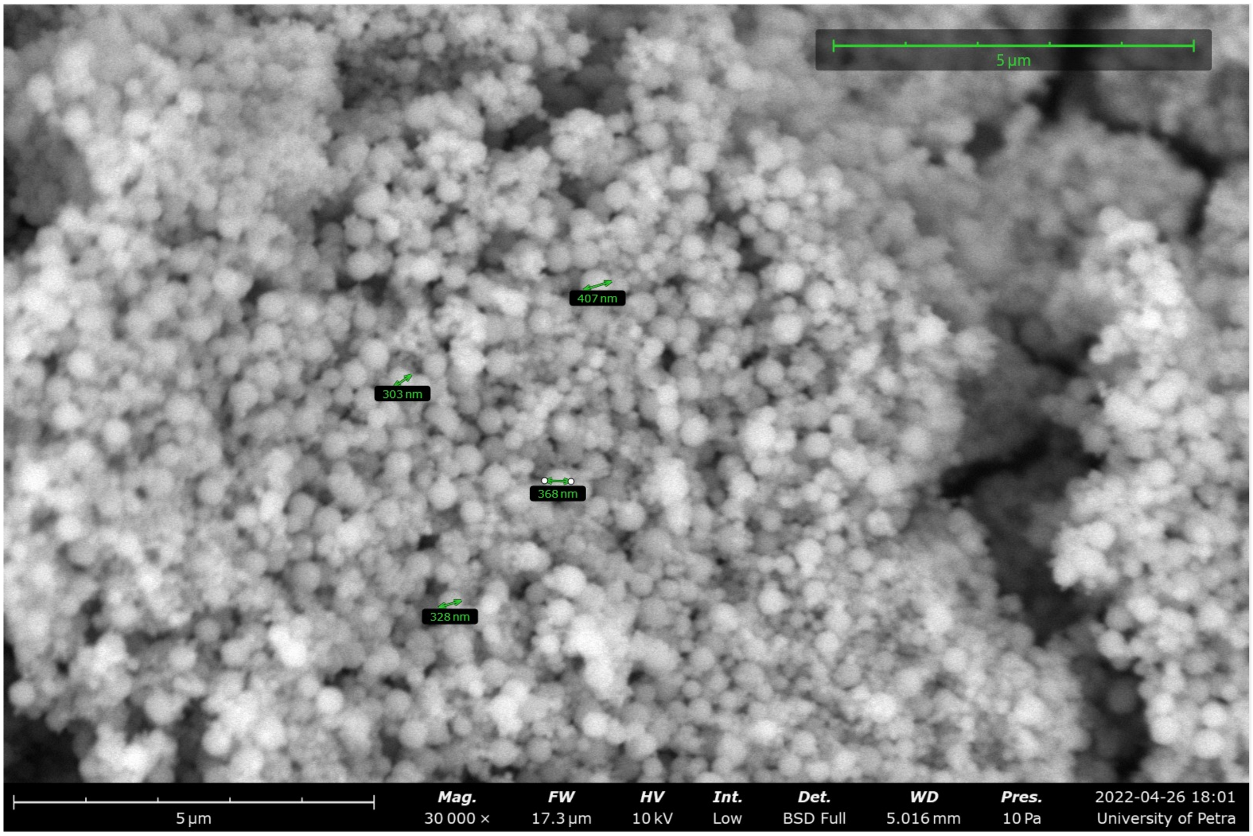

Supplementary Figure 24. SEM image of MOF-801. Scale bar is 5 μm.

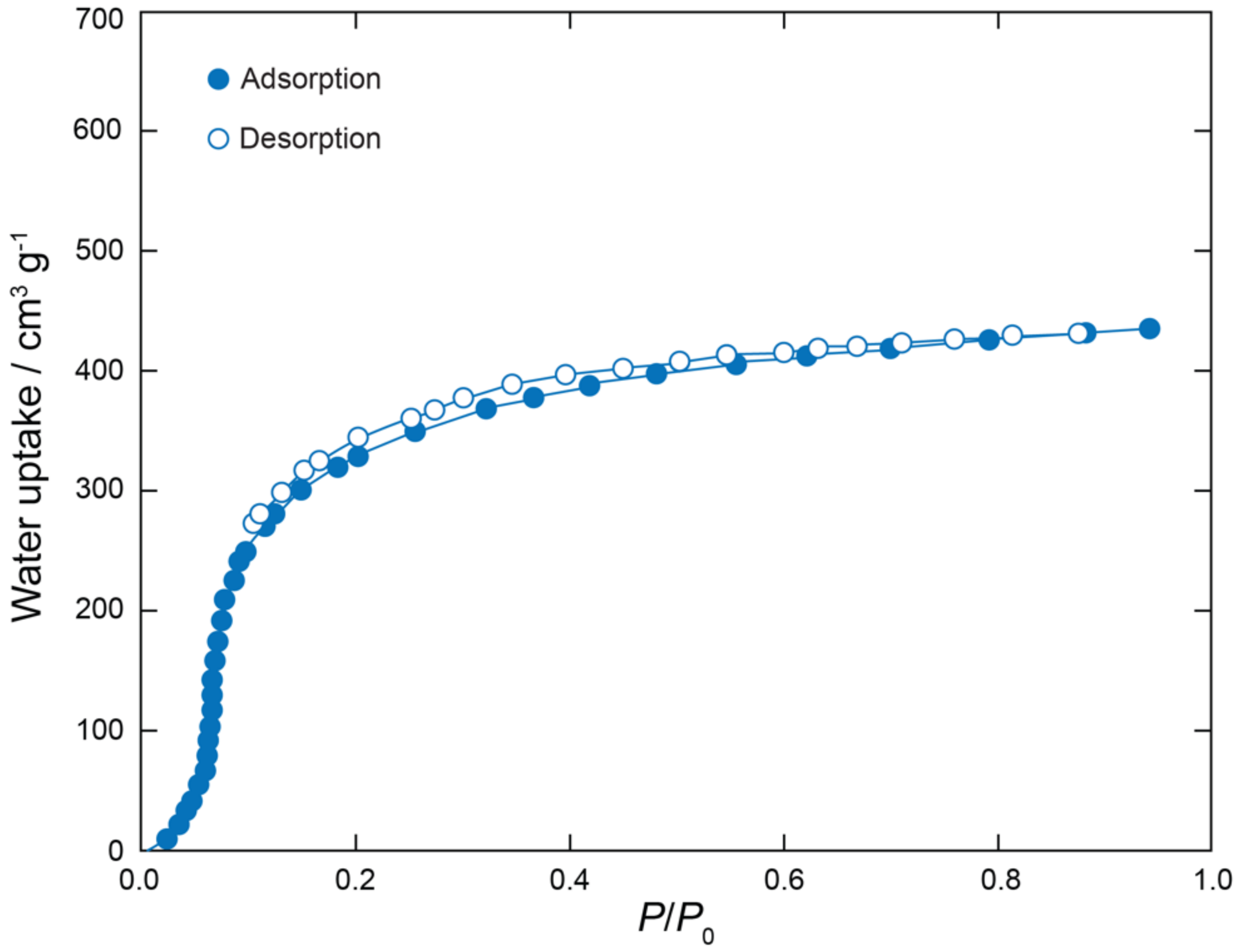

Supplementary Figure 25. Water sorption isotherm at 25 °C for MOF-801.[4]

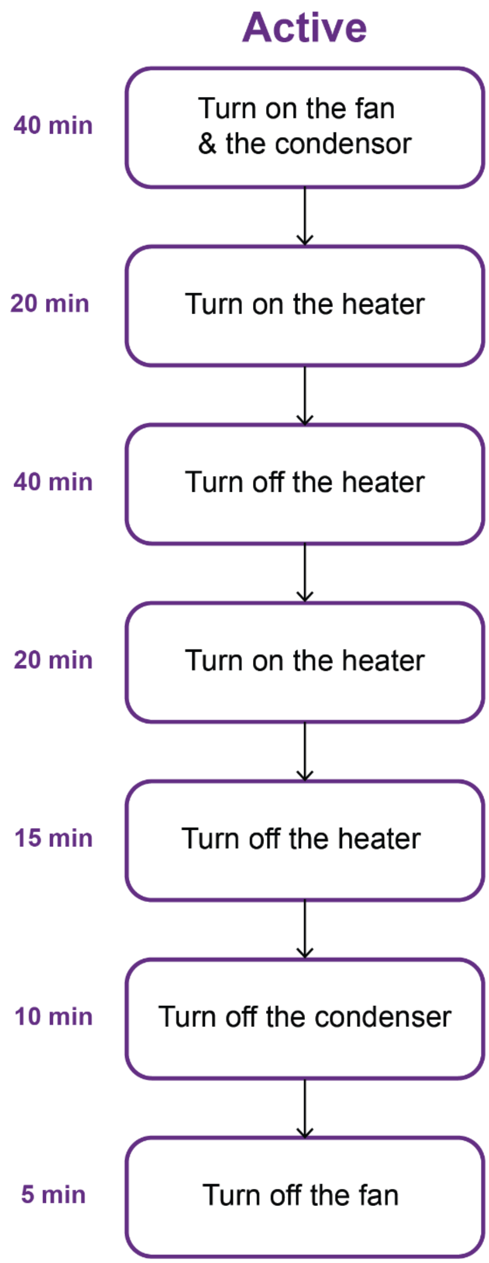

Supplementary Figure 26. Active mode of operation flow diagram.

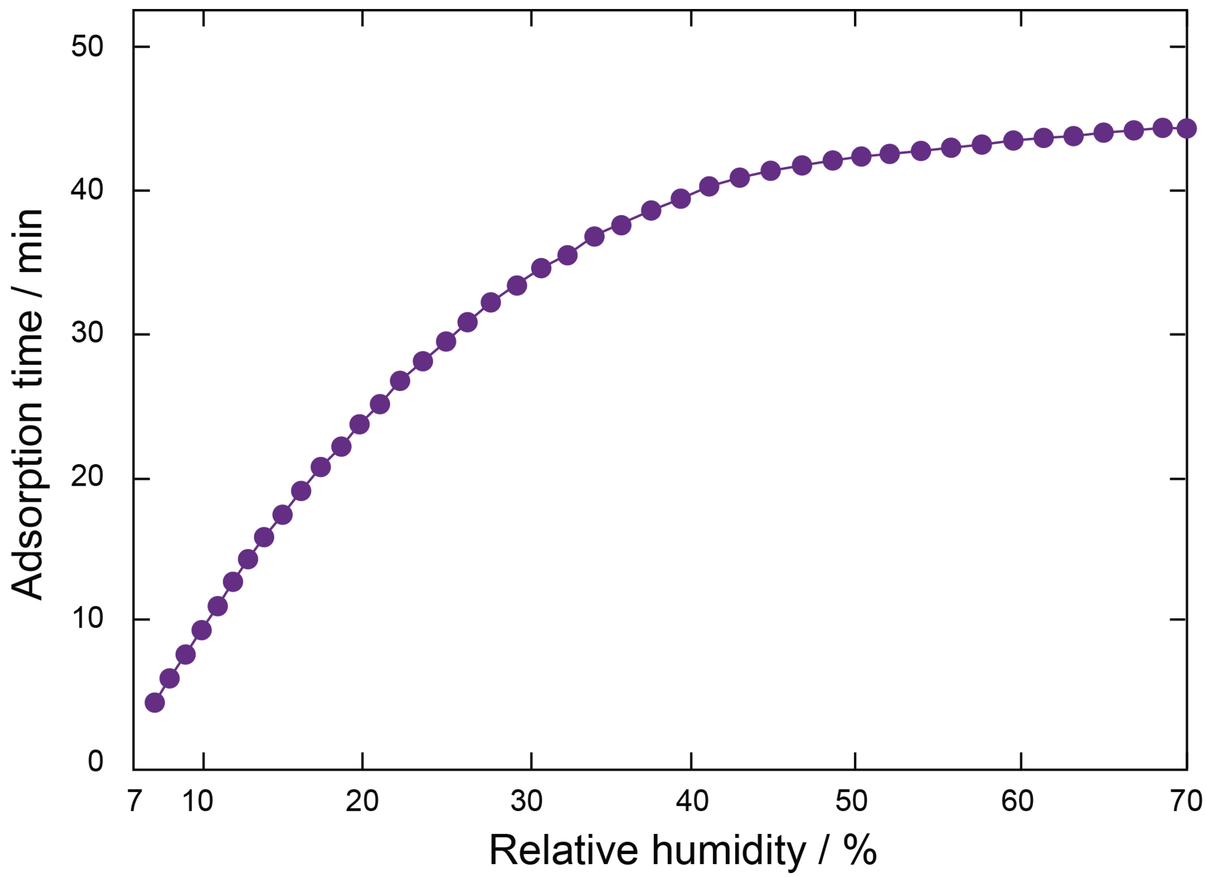

Supplementary Figure 27. The adsorption time in the adaptive mode of operation as a function of environmental RH.

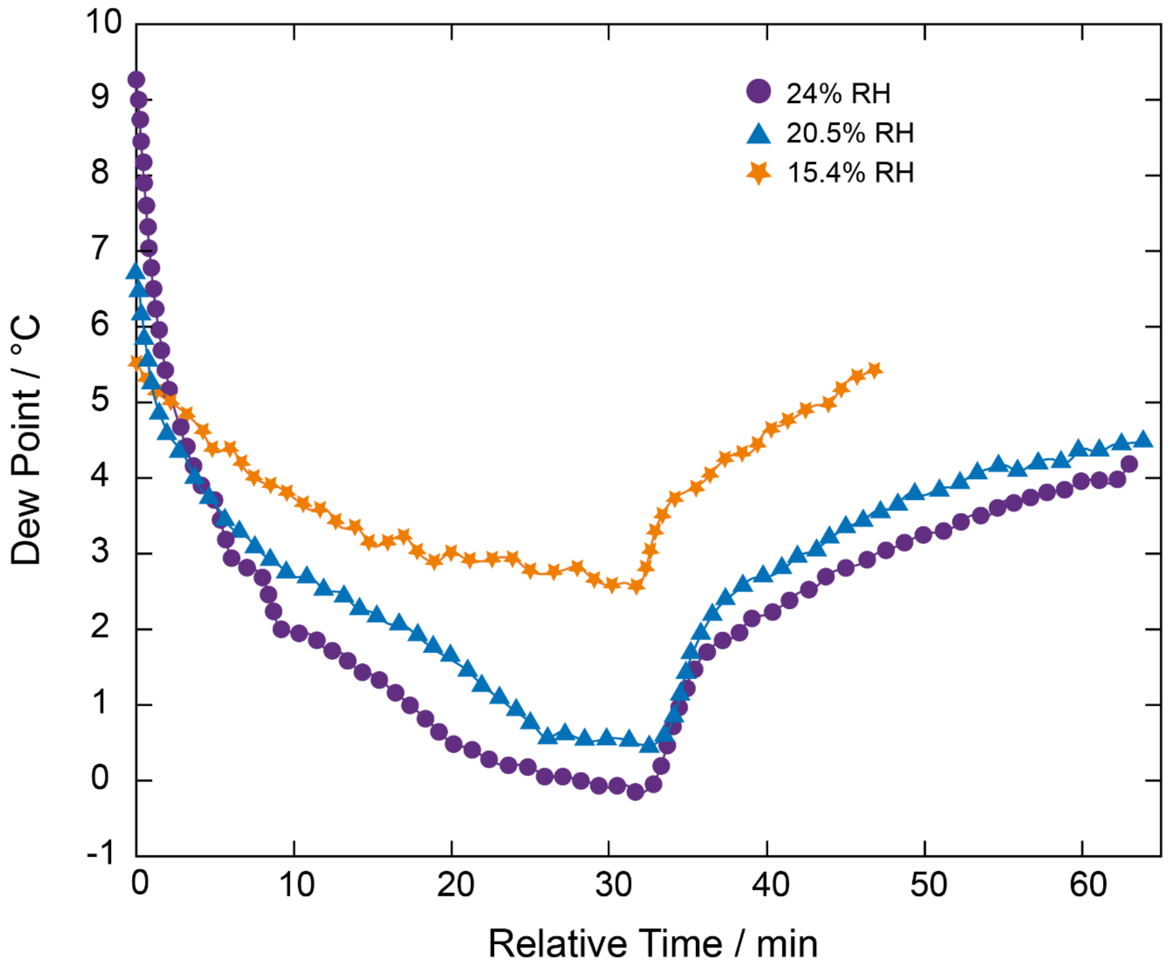

Supplementary Figure 28. Actual adsorption response at the condensation compartment after uploading the algorithms into the device for different climate conditions: 15.4 (orange stars), 20.5 (blue triangles), 24% RH (purple circles).

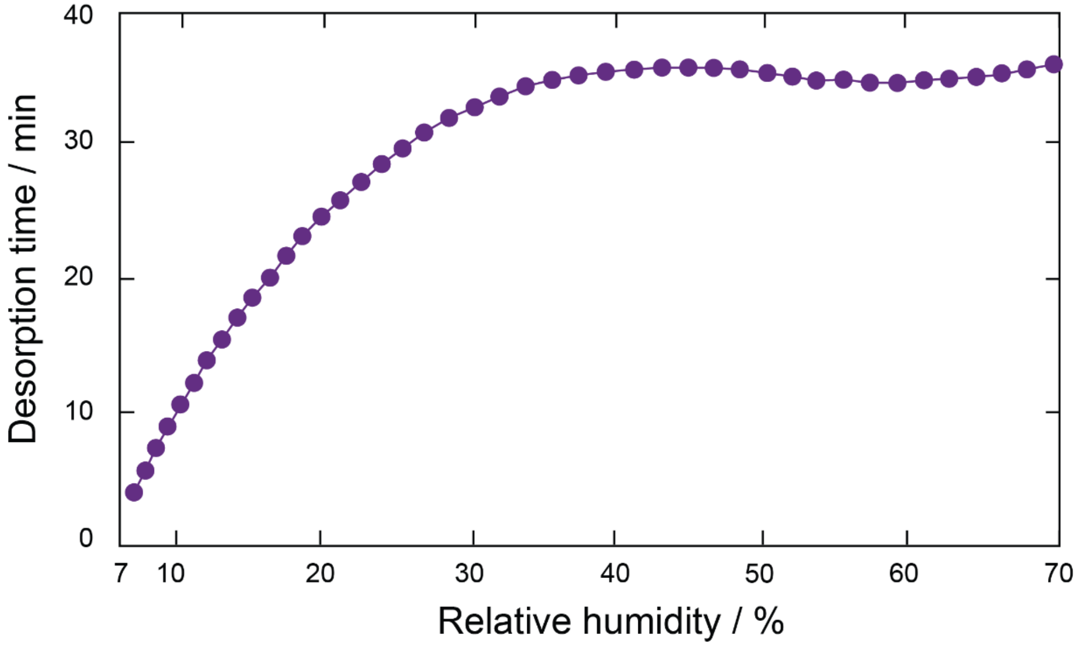

Supplementary Figure 29. The desorption time in the adaptive mode of operation as a function of environmental RH.

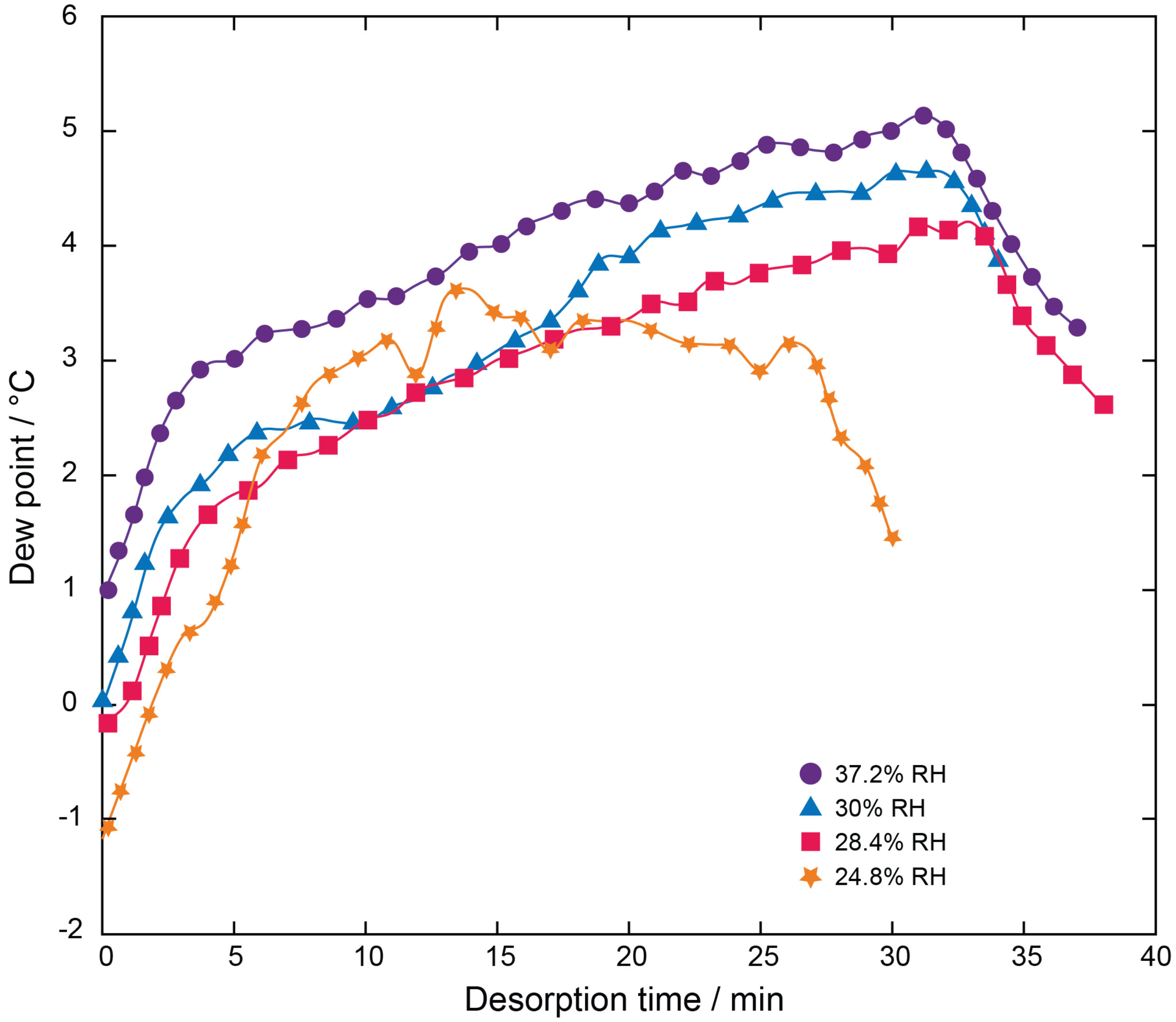

Supplementary Figure 30. Actual desorption response at the condensation compartment after uploading the algorithms into the device for different climate conditions: 24.8 (orange stars), 28.4 (pink squares), 30 (blue triangles), 37.2% RH (purple circles).

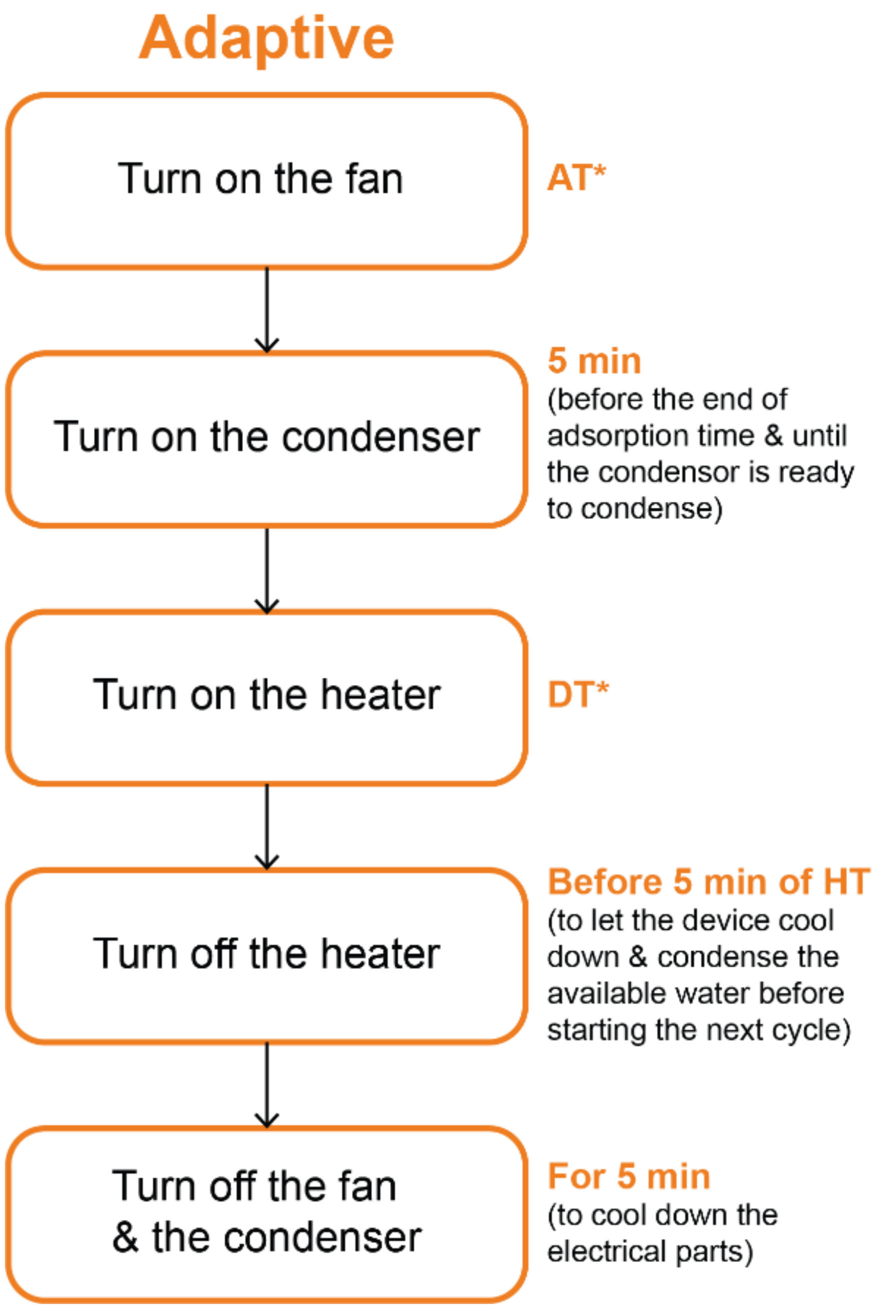

Supplementary Figure 31. Adaptive mode of operation flow diagram.

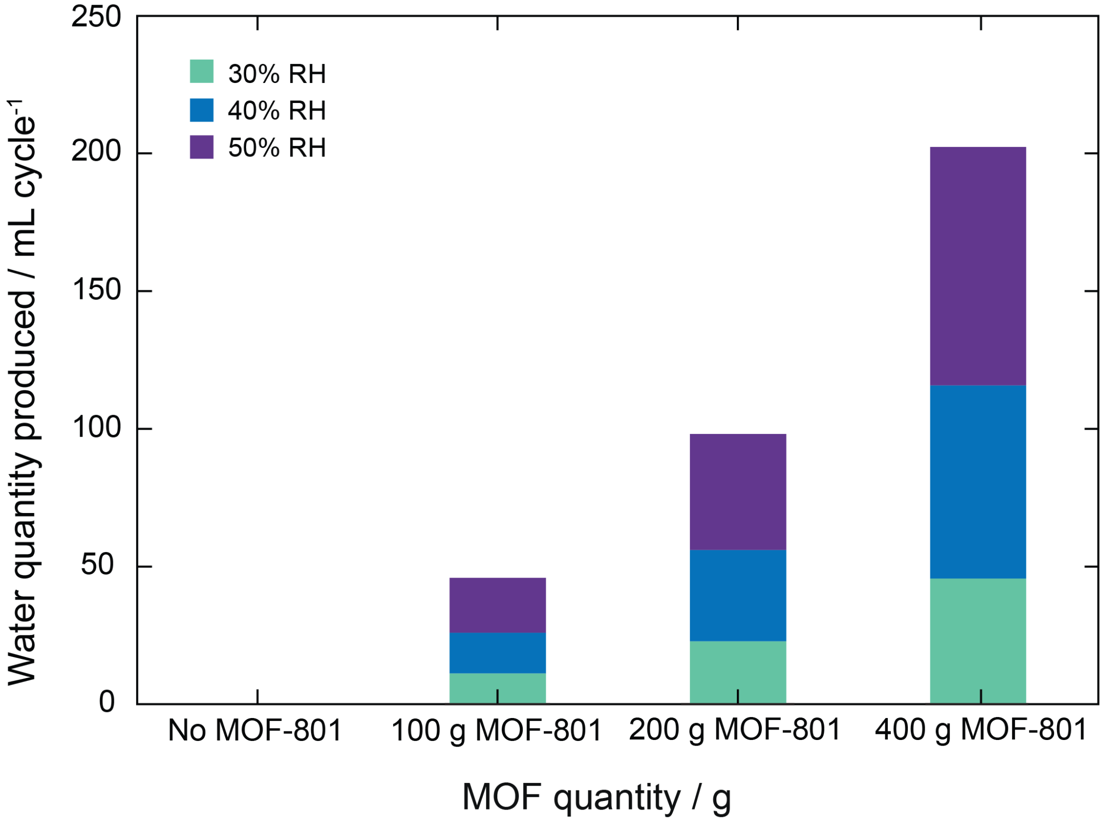

Supplementary Figure 32. Water productivity for the adaptive water harvester as a function of the amount of MOF-801 used in the device.

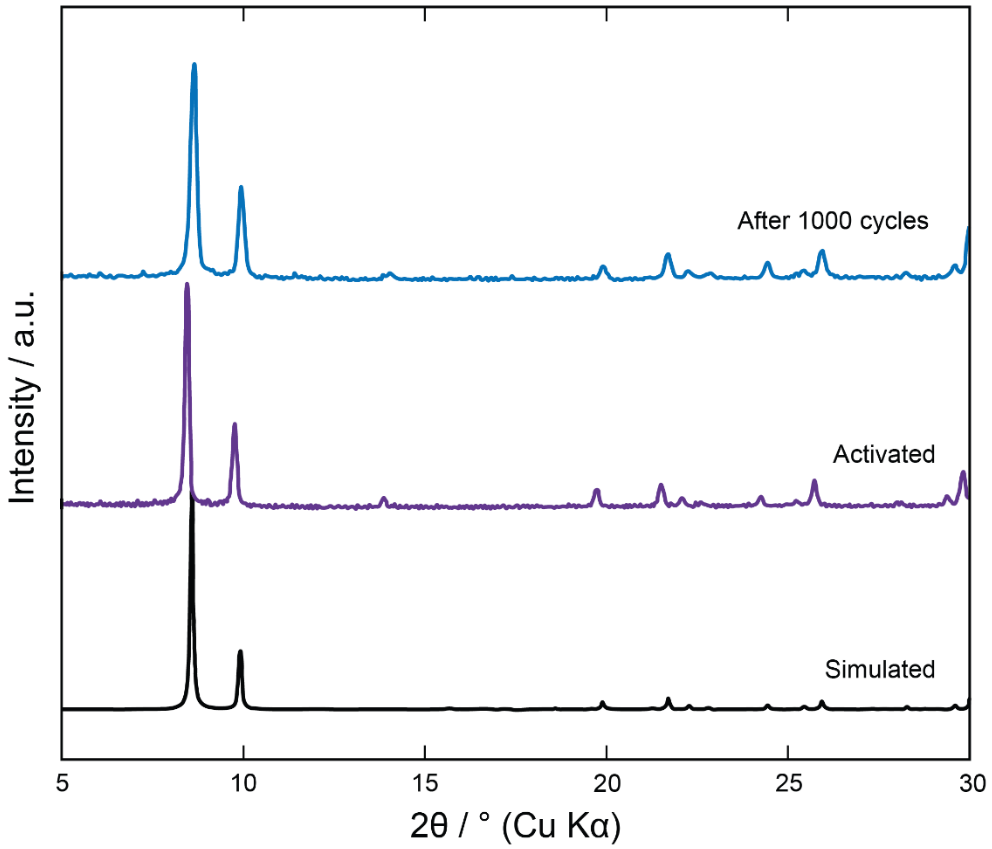

Supplementary Figure 33. PXRD pattern for simulated, activated and after 1000 cycles of using MOF-801 in the device (>1 year of use).

2. Supplementary Tables

Supplementary Table 1. Preliminary active device experiments.

Procedure description	Water produced at low RH (<20%)	Water produced at high RH (>20%)	Power consumption per L
**Procedure 1** 30 min of adsorption without activation of the condenser followed by 10 min of condenser pre-activation, then 30 min of desorption and 20 min with condenser off and, finally, 10 min of regeneration.	432 mL_{H2O} $kg_{MOF-801}^{-1}$ d^{-1}	658 mL_{H2O} $kg_{MOF-801}^{-1}$ d^{-1}	4.16 – 7.8 kWh L^{-1}
**Procedure 2** 40 min of adsorption phase, then 20 min of desorption phase and 15 min with condenser and heater off and, finally, 5 min of regeneration.	968 mL_{H2O} $kg_{MOF-801}^{-1}$ d^{-1}	1652 mL_{H2O} $kg_{MOF-801}^{-1}$ d^{-1}	2.36 – 8.1 kWh L^{-1}
**Procedure 3** 40 min of adsorption phase, then 20 min of desorption phase and then repeat another cycle with the same timing. Then, 15 min with heater off and, finally, 10 min of regeneration and 5 min of non-active fan.	1254 mL_{H2O} $kg_{MOF-801}^{-1}$ d^{-1}	2328 mL_{H2O} $kg_{MOF-801}^{-1}$ d^{-1}	3.506 – 7.87 kWh L^{-1}
**Procedure 4** 40 min of adsorption phase, then 20 min of desorption phase and then repeat another two cycles with the same timing. Then, 15 min with heater off and, finally, 10 min of regeneration and 5 min of non-active fan.	1583 mL_{H2O} $kg_{MOF-801}^{-1}$ d^{-1}	2574 mL_{H2O} $kg_{MOF-801}^{-1}$ d^{-1}	4.73 – 9.24 kWh L^{-1}

Supplementary Table 2. Power consumption under extreme conditions (40 ºC, 15% RH).

Electric components	Work duration per cycle (hr)	Power rating (W)	Energy consumption per cycle (Whr)	Produced water per cycle (mL kg_{MOF}^{-1})	Produced water per day (L kg_{MOF}^{-1})
Main fan	2.41	54.5	131.72	112.5	1.08
Compressor	2.25	184	414		
Compressor fan	2.25	5	11.25		
Heater	0.66	500	333.5		
Power consumption per L per d	**7.87 kWhr L^{-1} d^{-1}**				

Supplementary Table 3. Power consumption for average conditions (25 ºC, 60% RH).

Electric components	Work duration per cycle (hr)	Power rating (W)	Energy consumption per cycle (Whr)	Produced water per cycle (mL kg_{MOF}^{-1})	Produced water per day (L kg_{MOF}^{-1})
Main fan	2.417	54.5	131.72	252.5	2.424
Compressor	2.25	184	414		
Compressor fan	2.25	5	11.25		
Heater	0.667	500	333.5		
Power consumption per L per d	**3.506 kWhr L^{-1} d^{-1}**				

Supplementary Table 4. Time needed for the compressor to reach and maintain (for 20 min) the dew point value of 3.6 °C based on an ambient temperature of 22 °C.

Compression refrigeration cycle status	Time (min)
on	6:3
off	1:34
on	2:19
off	1:42
on	2:36
off	1:44
on	2:24
off	1:38
Total working time needed to maintain 3.6 °C at the condenser for 20 min	13.7 min

Supplementary Table 5. Time needed for the compressor to reach and maintain (for 20 min) the dew point value of 11.2 °C based on an ambient temperature of 22 °C.

Compression refrigeration cycle status	Time (min)
on	4:48
off	2:58
on	1:21
off	2:48
on	1:20
off	2:56
on	1:22
off	3:12
Total working time needed to maintain 11.2 °C at the condenser for 20 min	8.85 min

Supplementary Table 6. Comparison between the three main modes of operation for MOF-based water harvesters.

Mode of operation / comparison	Passive	Active	Active Reported Herein	Adaptive Reported Herein
Working principle	Single cycle / day	Multiple cycles/day	Multiple cycles/day	Multiple cycles/day+ environmental adaptation
Sorption Material requirements	Maximize material water capacity	Maximize material dynamic capacity	Maximize material dynamic capacity	Maximize material dynamic capacity + minimum inflection point
Working time and working environment	Produces water only on the day (desorption phase)	All day without consideration of variable environmental conditions	All day without consideration of variable environmental conditions	Works any time under any environmental condition
Continuous water production	No	Yes	Yes	Yes
Device Size needed to produce 3.5L/day	10.3 m^2 for single stage device. 4.54 m^2 for dual stage device.	Not available	0.32 m^2	0.32 m^2
Device Size needed to produce 25L/day	73.5 m^2 for single stage device. 32.4 m^2 for dual stage device.	Not available	0.32 m^2	0.32 m^2
Powered by	sun concentrators	PV panels	Electricity	Electricity
Water production in 10-30% relative humidity / kg of sorption material	0.25 L	0.7 L	1.2 L	1.8 L
Water production in 40-60% relative humidity / kg of sorption material	0.3 L	1.3 L	2.4 L	3.5 L
Number of cycles per day	1 cycle	9 cycles	18 cycles	Depends on the environmental conditions From 6 to >30 cycles daily
Automatic control	No	Yes	Yes	Yes
Power consumption/liter	-	-	3 – 7 kWh L^{-1}	1.67 – 5.25 kWh L^{-1}
Material used in the prototype	MOF-801	MOF-303	MOF-801	MOF-801
Water quality (filtration & mineralization)	No	No	Yes	Yes

Supplementary Table 7. Jordan’s national drinking standards water panel test for metals.

Type of Test	Unit (mg/L)	Results	Method Employed
Mo	mg/L	<0.01	SM 3120-B, 2017, (online)
As	mg/L	<0.01	SM 3120-B, 2017, (online)
Mn	mg/L	<0.05	SM 3120-B, 2017, (online)
Zr	mg/L	<0.1	SM 3120-B, 2017, (online)
Ag	mg/L	<0.1	SM 3120-B, 2017, (online)
B	mg/L	<0.1	SM 3120-B, 2017, (online)
Se	mg/L	<0.04	SM 3120-B, 2017, (online)
Ba	mg/L	<0.1	SM 3120-B, 2017, (online)
Cr	mg/L	<0.02	SM 3120-B, 2017, (online)
Ni	mg/L	<0.05	SM 3120-B, 2017, (online)
Na	mg/L	5.7	SM 3111-B, 2017, (online)
NO_3	mg/L	<1.0	SM 4110-B, 2017, (online)
SO_4	mg/L	<5.0	SM 4110-B, 2017, (online)
Hg	mg/L	<0.001	SM 3112-B, 2017, (online)
Pb	mg/L	<0.01	SM 3120-B, 2017, (online)
Cd	mg/L	<0.003	SM 3120-B, 2017, (online)
Al	mg/L	<0.1	SM 3120-B, 2017, (online)
Cu	mg/L	<0.05	SM 3120-B, 2017, (online)
Sb	mg/L	<0.02	SM 3120-B, 2017, (online)
Zn	mg/L	3.58	SM 3120-B, 2017, (online)
Fe	mg/L	<0.1	SM 3120-B, 2017, (online)

Supplementary Table 8. Jordan's national drinking standards water panel tests for volatile organic compounds.

Type of Test	Unit (µg/L)	Results	Method Employed
Benzene	µg/L	<10.0	SOP No. 71/02/01/01-23 Issue 1
Total Xylene	µg/L	<20.0	SOP No. 71/02/01/01-23 Issue 1
Trichloroethene	µg/L	<20.0	SOP No. 71/02/01/01-23 Issue 1
Tetrachloroethene	µg/L	<20.0	SOP No. 71/02/01/01-23 Issue 1
Ethylbenzene	µg/L	<20.0	SOP No. 71/02/01/01-23 Issue 1
Toluene	µg/L	<20.0	SOP No. 71/02/01/01-23 Issue 1

Supplementary Table 9. Jordan's national drinking standards water panel test for microbiology.

Type of Test	Unit (µg/L)	Results	Method Employed
Pseudomonas aeruginosa	CFU/100mL	Less than 1	SM 9213 E: 2017, online
Fecal Streptococci	MPN/100mL	Less than 1.10	SM 9230 B: 2017, online
Fecal Enterococcus	MPN/100mL	Less than 1.10	SM 9230 B: 2017, online
Algae type and count	Cells or units/mL	Not seen	SM 10200:2017, online
Heterotrophic plate count	CFU/mL	8.3×10^2	SM 9215 AB: 2017, online

Supplementary Table 10. Cost summary for 1 kg device without electricity running cost.

Category	Amount needed*	Price ($) **	Total Price	% Total Price	Notes
Raw Chemical Costs	1	164.926	164.926	31	
Material Costs	1	315.36	315.36	46	
Capital (One-Time) Costs***	0.067	1043.71	69.92857	14	
Labor Costs	1	77	77	9	
**Total production cost per unit ($, USD)**			**625**		
**Cost per L of water produced ($, USD)**			**0.064**	10-year lifespan and 2.65 L H_2O production/device/day	
**Device cost per day of use ($, USD)**			**0.17**	Same assumptions	
*Calculated based on the production of 100 kg of porous material for use in 100 individual units					
**Calculated as price contribution per unit					
***Depreciation expense at 7% annually					

Supplementary Table 11. Device construction assuming 100 units of production.

Type	Amount needed*	Price per unit ($, USD)	Total Price	Source
Vapor-compression tech	100	105.75	10575	Local
Fan	100	32.43	3243	Local
Heating coil	100	42.3	4230	Local
Collection flask	100	28.2	2820	Local
Filters	100	14.1	1410	Local
Body	100	80.6	8060	Local
Sensors & controllers	100	82.4	8240	Local
Screws and sealing	100	30.7	3070	Local
Misc. parts & supplies	1	400	400	Local
**Total ($, USD)**			**42048**	
**Material price per unit ($, USD)**			**420.48**	
**Estimated bulk material price per unit ($, USD)**			**315.36**	
*Calculated for production of 100 kg of porous material for use in 100 individual units				

Supplementary Table 12. Raw chemical costs assumed production of 100 kg of porous material.

Chemical	Amount needed*	Price per unit ($, USD)	Total Price	Source
Zirconyl chloride	160 kg	14.20/kg for 50 kg	2272	Infratmat, US
Fumaric acid	58 kg	18.2/kg for 10 kg	1055.6	Fisher Scientific
DMF	500 L	12/L for 200 L	6000	Sigma Aldrich
Acetic acid	7000 L	0.94/kg for MT	6580	Echemi
Methanol	1500 L	0.39/L for MT	585	Global market price
**Total ($, USD)**			**16492.6**	
**Chemical cost per kg of porous material produced ($, USD)**			**164.926**	

Supplementary Table 13. Laboratory equipment capital costs assuming production of 100 kg of porous material.

Supply	Amount needed*	Price per unit ($, USD)	Total Price	Source
Reaction Bottles (10L)	500	100	5000	Wheaton, Corning, Pyrex
Isothermal Oven	10	3338	33380	Fisher Scientific
Precision Balance	1	991	991	Mettler-Toledo
Centrifuge	1	25000	25000	Thomas Scientific
Rotovap	1	20000	20000	Buchi
Misc. glassware	1	15000	15000	Funnels, spatula, centrifuge tubes
Misc. consumables	1	5000	5000	Waste containers, recycling distillation set-up, gloves
**Total**			**104371**	
**Total per unit ($, USD)**			**1043.71**	
*Calculated for production of 100 kg of porous material for use in 100 individual units				

Supplementary Table 14. Labor costs based on Jordanian rates.

**Labor Costs***			
Employee Type	**Hours needed****	**Price per hour ($, USD)**	**Total Price**
Chemical Engineer	350	5	1750
Chemist	700	3.5	2450
Mechanical Engineer	700	5	3500
**Total**			**7700**
**Total per unit ($, USD)**			**77**
*Large scale synthesis process requires 7 h of work per kg produced			
**Calculated for production of 100 kg of porous material for use in 100 individual units			

Supplementary Table 15. Cost per liter for 1 kg device with on-grid and off-grid electricity running cost.

**Country**	**Population without MSDW %**	**Commercial drinking water price ($, USD)** [6]	**Household electrical energy cost ($/kWh)** [7]	**Adaptive AWH drinking water cost ($/L)**	**Highest price reduction %**
Jordan – off grid	11 – 20	0.42	0.1	0.064	87.7
Jordan – on grid	11 – 20	0.42	0.1	0.167 – 0.525	45.71

MSDW: safely managed drinking water

3. Supplementary Notes

Supplementary Note 1. Design and construction of the water harvesting device

The adaptive atmospheric water harvesting device constructions includes three compartments, the air intake compartment (Supplementary Figure 1-5), the sorption compartment (Supplementary Figure 6-13), the condensation compartment (Supplementary Figure 14-18). The final device assembly is constructed by assembling the three compartments on a common base (Supplementary Figure 19-20).

Supplementary Note 2. MOF-801 synthesis and characterization

Material and supplies for MOF-801 synthesis. For MOF-801 synthesis, zirconyl chloride octahydrate ($ZrOCl_2.8H_2O$; purity ≥98%), zirconium(IV) chloride anhydrous ($ZrCl_4$; purity ≥98%), and fumaric acid (purity ≥99%) were purchased from Acros. Formic acid (purity 99%), methanol (purity ≥99.8%), *N*,*N*-dimethylformamide (DMF; purity ≥99.8 %) were purchased from either Carlo Ebra, Honeywell or Fisher Chemicals.

MOF-801 Synthesis. MOF-801 was synthesized by one of four different ways, with each way differing from each other in the source of the metal and the solvent used.

Procedure 1. MOF-801 was prepared by weighing of zirconyl chloride octahydrate (16 g, 50 mmol) and fumaric acid (5.8 g, 50 mmol) in a 500 mL glass bottle then dissolving these solids by adding a mixture of solvents formic acid (70 mL) DMF (200 mL) with stirring to obtain a clear solution. Then, the bottle was closed tightly and placed in the oven at 130 °C for 24 h. The resulting white precipitate (11.9 g) was separated from the mother liquid by decantation. 200 mL of DMF was used to wash the precipitate 3 times per day for 2 days, followed by solvent exchange with 200 mL of methanol 3 times daily for two days[1]. The precipitate was transferred by adding new methanol (50 mL) to a 100 mL round bottom flask, which was refluxed to activate the material. This process was repeated twice and solid was extracted by filter paper. The powder was dried in the air and then heated at 85 °C for two days (10.3 g,91% yield).

Procedure 2. zirconium (IV) chloride anhydrous (11.7 g, 50mmol) and fumaric acid (5.8 g,50 mmol) were weighed in a 500 mL glass bottle and then dissolved in a mixture of solvents formic acid (70 mL) and DMF (200 mL) with stirring to obtain a clear solution. Then, the bottle was closed tightly and placed in the oven at 130 °C for 24 h. The washing,

solvent exchanging, and activation procedure was the same as Procedure 1 to get 9.9g, 82% yield.

Procedure 3. zirconyl chloride octahydrate (1.3 g,4.13 mmol) and fumaric acid (1.44 g,12.39 mmol) were weighed in a 500 ml glass bottle then dissolved in a mixture of solvents containing formic acid (15.6 mL) and distilled water (80 mL). The mixture was sonicated for 120 minutes until the color of the solution becomes milky white and then the bottle was placed in the oven at 130 °C for 24 hours. The precipitate was separated by centrifugation and water was added to wash the isolated precipitate 3 times per day for 3 days followed by solvent exchange with methanol 3 times per day for three days [2]. The precipitate was transferred by adding new methanol (50 mL) to a 100 mL round bottom flask, which was refluxed to activate the material. This process was repeated twice. The powder was dried in a vacuum oven at 100 °C for 24 hours (1.2g, 95% yield).

Procedure 4. zirconium (IV) chloride anhydrous (0.96 g,4.13 mmol) and fumaric acid (1.44 g,12.39 mmol) were weighed in a 500 ml glass bottle and then dissolved in a mixture of solvents containing formic acid (15.6 mL) and distilled water (80 mL). [2] The washing, solvent exchanging, and the activation procedure was the same as Procedure 3 (0.8g, 92% yield).

MOF-801 characterization techniques. PXRD. Powder X-ray diffraction (PXRD) diffraction experiments were collected at the MS beamline of the SESAME synchrotron (Supplementary Figure 21). MOF-801 powder was filled in 0.5 mm quartz capillary and a full scan from 5 to 50 degrees was collected (an image every 5-degree step) measured at 15 keV with 20 s acquisition time at each detector frame. Every detector frame covers about 6.4 degrees, and five degrees from each frame was extracted by integrating the image along the beam center and then converting the pixels to corresponding two theta

angles as obtained from Si 640f (NIST) standard measurement. The images processing steps were done by an in-house macro script through image J software that create a data file for each image covering five degrees, and these data files were then merged using an in-house python script [3].

Porosity and surface area. A micromeritics Gemini VII 2390 Series gas adsorption analyzer was used to measure N_2 adsorption isotherms at 77 K for activated MOF-801. The BET was calculated from the data ranging from 0.01 to 0.8 and the result ranged from 370 to 649 $m^2\ g^{-1}$ depending on the sample and synthetic procedure used (Supplementary Figure 22).

Fourier-transform infrared spectroscopy (FT-IR). FT-IR spectroscopy was performed on a Shimadzu IR-Prestige-21 in transmittance mode over wavenumber range: 400-4000 cm^{-1} (Supplementary Figure 23).

Scanning electron microscopy (SEM). SEM imaging was performed using a Thermo Fisher Scientific Phenom XL G2 scanning electron microscope. The SEM images were collected at 10^{-4} Pa and with 15 kV accelerating voltage. (Supplementary Figure 24).

Supplementary Note 3. Active water harvesting measurements

Preliminary and optimized active device timing. For active water harvesting, the device was tested over 10 – 70% RH and 15 – 35 °C with WHC_{active} timings. This sequence was concluded from timing 4 processes, each one is an improvement of the previous one based on visual indications of water production with time. Supplementary Table 1 is a summary of those experiments. (Supplementary Figure 26). (Supplementary Table 1-3).

Reduced power consumption when MOF is used. The purpose of using MOFs in the water harvesting device is to concentrate the water quantity in the air, which subsequently increases the dew point (the temperature that the condenser is required to reach to condense the water in the air) at the condensation compartment. By understanding and exploiting this fact, we can reduce the power consumed by the compression refrigeration cycle. To study the time needed to reach 3.6 °C, the compression refrigeration cycle (189 W power rating) was working according to the times detailed in Supplementary Table 4. To reach 11.2 °C, the compression refrigeration cycle was working according to the times detailed in Supplementary Table 5. According to this data, the compressor power consumption is reduced by 55% when MOF-801 was used.

Dew point calculation. The distributed data loggers measured, in real-time, the temperature and the relative humidity for the air in the different compartments. For the inlet air, the dew point then is calculated using Supplementary Equation 1.[5]

$$DP = \frac{B_1[\ln\left(\frac{RH}{100}\right) + \frac{A_1 T}{B_1 + T}]}{A_1 - \ln\left(\frac{RH}{100}\right) - \frac{A_1 T}{B_1 + T}}$$

Supplementary Equation 1. Calculating dew point temperature (DP) using the relative humidity (RH) and the temperature of the air (T), where A_1 and B_1 are constants and equal to 17.625 and 243.04 respectively.

Supplementary Note 4. Adaptive water harvesting measurements

Adaptive adsorption phase. The preliminary experiments for the previous active mode of operation were used to adapt the new mode of operation to changes in the climate conditions (Supplementary Figure 27-28). The adsorption time needed to saturate the material differs based on the change in climate conditions and from this data an algorithm was developed (Supplementary Equation 2).

$$\mathbf{AT = 0.0002(RH)^3 - 0.0378(RH)^2 + 2.4714(RH) - 11.257 \qquad R^2 = 0.9983}$$

Supplementary Equation 2. An algorithm was developed based on adsorption experiments to calculate the appropriate adsorption time for the device in climate conditions ranging from the inflection point (RH = 7%) to the very high RH = 70%. This algorithm is developed for MOF-801.

Adaptive desorption phase. Preliminary experiments using the previous active mode of operation were used to adapt the new mode of operation to changes in climate conditions. The desorption time needed to regenerate the material differs based on changes to the climate conditions. (Supplementary Figure 29-31).

$$\mathbf{DT = (500/Hp)\ (0.004(RH)^3 - 0.0621(RH)^2 + 3.1302(RH) - 15.625) \qquad R^2 = 0.9995}$$

Supplementary Equation 3. An algorithm was developed based on preliminary desorption experiments to calculate the appropriate desorption time for the device over a climate condition ranging from the inflection point of RH = 7% to the very high RH = 70%, where Hp is the used electric heater power in Watts. This algorithm is for MOF-801.

Supplementary Note 5. Performance evaluation

A comparison between the main three modes of operation is done, see Supplementary Table 6.

MOF-801 quantity effect on water production. A control experiment was conducted under multiple climate conditions (20, 30, 40% RH) with a gradual increase in the quantity of MOF-801 used in the device (0, 100, 200, 400 g of MOF-801). We found that the water productivity of the adaptive device is directly dependent on the MOF quantity loaded in the device. (Supplementary Figure 32).

Supplementary Note 6. Long-term performance

To harvest water from air throughout the year the MOF material should retain performance without losing its crystallinity. Supplementary Figure 33 shows the PXRD for MOF-801 after 1000 cycles (>1 year of operation). PXRD measurements were carried out using a Rigaku MiniFlex 600 that has a sealed-tube copper X-ray source (λ = 1.5418 Å), a scintillation counter with high dynamic range, and Bragg-Brentano geometry with slits providing high resolution for flat powder samples. A sample was placed on a zero-background sample holder and the data was collected scan range from 5 to 30 degrees with scan step of 0.05 and scan speed 3.0 for all samples.

Supplementary Note 7. Full water analysis

A full water analysis was done. For metals test see Supplementary Table 7, volatile organic compounds see Supplementary Table 8, microbiology see Supplementary Table 9.

Supplementary Note 8. Cost analysis

a full cost analysis was conducted for the adaptive atmospheric water harvesting device. A summary in Supplementary Table 10, device construction cost breakdown Supplementary Table 11, raw chemicals cost Supplementary Table 12, laboratory equipment capital cost Supplementary Table 13, labor costs Supplementary Table 14, on grid and off grid cost Supplementary Table 15.

4. Supplementary References

1. Fathieh, F.; Kalmutzki, M. J.; Kapustin, E. A.; Waller, P. J.; Yang, J.; Yaghi, O. M. Practical Water Production from Desert Air. *Sci. Adv.* **4**, eaat3198 (2018).

2. Jahan, I.; Rupam, T. H.; Palash, M. L.; Rocky, K. A.; Saha, B. B. Energy efficient green synthesized MOF-801 for adsorption cooling applications. *J. Mol. Liq.* **345**, 117760 (2020).

3. Abdellatief, M.; Rebuffi, L.; Khosroabadi, H.; Najdawi, M.; Abu-Hanieh, T.; Attal, M.; Paolucci, G. *Powder Diffr.* **32**, S6-S12 (2017).

4. Furukawa, H.; Gandara, F.; Zhang, Y.-B.; Jiang, J.; Queen, W. L.; Hudson, M. R.; Yaghi, O. M. Water Adsorption in Porous Metal–Organic Frameworks and Related Materials. *J. Am. Chem. Soc.* **136**, 4369-4381 (2014).

5. Lawrence, M. G. The relationship between relative humidity and the dewpoint temperature in moist air: A simple conversion and applications. *Bull. Amer. Met. Soc.* **86(2),** 225-234 (2005).

6. Jordan - Bottled water - price, March 2022 | GlobalProductPrices.com (Accessed June 27, 2022).

7. Jordan electricity prices, September 2021 | GlobalPetrolPrices.com (Accessed June 27, 2022).
